# The relationship between *APOE* genotype, CSF Tau and cognition across the Alzheimer's disease spectrum, moderation and mediation role of insula network connectivity

**DOI:** 10.1111/cns.14401

**Published:** 2023-08-14

**Authors:** Yao Zhu, Yan Wu, Xinyi Lv, Jiaonan Wu, Chunzi Shen, Qiqiang Tang, Guoping Wang, Alzheimer's Disease Neuroimaging Initiative

**Affiliations:** ^1^ Department of Neurology, The First Affiliated Hospital of USTC, Division of Life Sciences and Medicine University of Science and Technology of China Hefei China

**Keywords:** Alzheimer's disease, apolipoprotein E, insula network, mediation analysis, moderation analysis

## Abstract

**Aims:**

To investigate whether insula network connectivity modulates the relationship between apolipoprotein E (*APOE*) ε4 genotype, cerebrospinal fluid (CSF) biomarkers (Aβ, Tau, and pTau) and cognition across Alzheimer's disease (AD) spectrum.

**Methods:**

Forty‐six cognitive normal (CN), 35 subjective memory complaint (SMC), 41 mild cognitive impairment (MCI), and 32 AD subjects from the Alzheimer's Disease Neuroimaging Initiative (ADNI) were obtained. Multivariable linear regression analyses were conducted to investigate the main effects and interaction of the *APOE* genotype and disease status on the insula functional connectivity (IFC) network. Mediation and moderation analysis were performed to investigate whether IFC strengths regulate the association between *APOE* genotype, CSF biomarkers and cognition. Additionally, the support vector machine (SVM) model integrating *APOE* genotype, CSF biomarkers, and neuroimaging biomarkers (insula volumes and altered regional IFCs) was used to classify the AD spectrum.

**Results:**

The interactive effect of the *APOE* genotype and disease on the insula network was found in the left medial superior frontal gyrus (SFGmed.L), right anterior medial prefrontal cortex (aMPFC.R), and bilateral thalamus (THA.B). The functional connectivities (FCs) in the left insula (LIns) connecting with the left posterior middle temporal gyrus (pMTG.L), SFGmed.L, and right lingual gyrus (LING.R) were correlated with cognition. LIns‐SFGmed.L and LIns‐pMTG.L FCs could moderate the effects of Tau on cognition. Furthermore, LIns‐SFGmed.L FC may suppress the association between *APOE* genotype and cognition. More importantly, the integrated biomarkers from the SVM model yielded strong powers for classifying the AD spectrum.

**Conclusions:**

Insula functional connectivity regulated the association between *APOE* genotype, CSF Tau and cognition and provided stage‐dependent biomarkers for early differentiation of the AD spectrum. The present study used a cross‐sectional design. Follow‐up studies are needed to validate the relationship.

## Graphical abstract

Statistical diagrams present the effect of Tau, LIns‐SFGmed.L connectivity, and interaction of Tau with LIns‐SFGmed. connectivity on cognition across the AD spectrum. Mediation analysis revealed that the LIns‐SFGmed.L connectivity mediated the relationship between the *APOE* genotype and cognition across the AD spectrum.

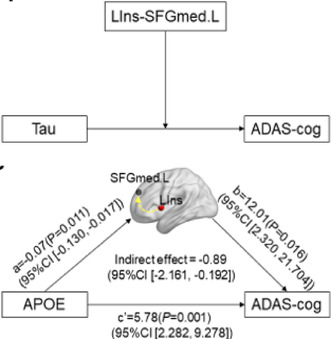

## 1 INTRODUCTION

Alzheimer's disease (AD) is a disabling neurodegenerative disease characterized by the loss of synapses and neurons and the decline of neuronal connections. The AD spectrum ranges from cognitive normal (CN) to subjective memory complaint (SMC), to mild cognitive impairment (MCI) and AD dementia.^1^, ^2^ Amyloid deposition, pathologic tau, and neurodegeneration (ATN) Biomarkers used neuroimaging (PET and MRI) and cerebrospinal fluid (CSF) to to classify AD spectrum populations.^3^ Increased concentration of CSF Tau might reflect the degree of neuronal degeneration. A large body of literature demonstrated an early plateauing of CSF Tau and p‐Tau in pre‐symptomatic AD individuals.^4^ CSF Tau showed a prominent increase early on, in the preclinical stage of AD when only minimal Aβ‐pathology has been detected.^5^ Resting‐state functional magnetic resonance imaging (rs‐fMRI) is considered an effective method to analyze complex neural network by measuring the inherent brain fluctuations of blood oxygen level‐dependent (BOLD) signals.^6^, ^7^ AD is likely influenced by genetic and environmental risk factors. *APOE* ε4 is the strongest genetic risk factor for AD, which might enhance AD pathology and cognition dysfunction.^8^, ^9^

Previous studies suggested that the APOE genotype might modulate cognitive performance through the brain networks.^10^, ^11^ It is reported that the existence of disrupted FCs within neural networks such as The default mode network (DMN), the executive control network (ECN), the salience network (SN) and sensorimotor network (SMN) in AD patients.^12^ The insula is connected with fronto‐parietal regions, and temporal sulcus, primary sensory and motor cortices, as well as the anterior cingulate cortex.^13^ The insula is one site affected by early pathological changes in AD and is a crucial hub of the human brain networks.^14^ Previous studies have shown that insula gray matter (GM) loss in AD patients, abnormal local insula activity in MCI subjects, and disrupted insula network connectivities play an important role in episodic memory impairment.^15^, ^16^, ^17^ However, the effect of insula functional connectivity (IFC) in regulating the association between APOE genotype, CSF biomarkers, and cognition appears to be unknown across the AD spectrum. These strongly support the use of the insula as a seed region for rs‐fMRI studies.

The purposes of this study were to assess the insula GM volume changes, and whether the IFC regulated the associations between *APOE* genotype, CSF biomarkers, and cognition across AD spectrum subjects. More importantly, we used the support vector machine (SVM) model integrating *APOE* genotype, CSF biomarkers (Aβ, Tau, and pTau), and neuroimaging biomarkers (insula volumes and altered regional IFCs) to classify AD spectrum and evaluate classifier performance.

## 2 MATERIALS AND METHODS

### 2.1 ADNI database and participants

All data were obtained from the Alzheimer's disease Neuroimaging Initiative (ADNI) database (http://adni.loni.usc.edu). The rs‐fMRI scan images were all from ADNI‐1, ADNI‐GO, ADNI‐2, and ADNI‐3 phases. If two or more rs‐fMRI scans were performed at baseline, the first available scan was included for analysis. Corrupted image sequences, image quality control failure, and excessive head motion were excluded. 46 cognitive normal (CN), 35 SMC, 41 MCI, and 32 AD subjects were included. Individuals with one or more *APOE* ε4 alleles were classified as *APOE* ε4 carriers (*APOE* ε4+), while subjects with no *APOE* ε4 alleles were classified as *APOE* ε4 non‐carriers (*APOE* ε4−). All subjects underwent a battery of cognitive evaluations, including global cognitive function (Mini‐Mental State Examination [MMSE] and Alzheimer's Disease Assessment Scale–13‐item cognitive subscale [ADAS‐cog]) and episodic memory function (the Rey Auditory Verbal Learning Test [RAVLT], the RAVLT‐immediate [RAVLT‐I] reflects the total acquisition/learning, the RAVLT learning [RAVLT‐L] reflects the learning rate, the RAVLT forgetting [RAVLT‐F] reflects the long‐term retention). The diagnostic criteria, neuropsychological assessment, CSF biomarkers, image acquisition parameters, and image processing were presented in the Appendix S1.

### 2.2 Statistical analysis

#### 2.2.1 Demographic information and neuropsychological performance

The Shapiro–Wilk test was adopted to assess the data normality of continuous variables. Levene's test was examined to assess the homogeneity of variance. Non‐parametric Kruskal‐Wallis test was used if the Shapiro–Wilk test or Levene's test *p* < 0.05. Chi‐square tests were used to compare the gender. Mixed analysis of covariance (ANCOVA), with the disease status and *APOE* genotype as fixed factors, was used to analyze the neuropsychological and demographic data among subjects with statistically significant differences (*p* < 0.05), followed by post hoc test to determine the significance of the specific comparisons. All statistical procedures utilized SPSS 25.0 software (SPSS, Inc.).

#### 2.2.2 Insula network analysis

To analyze the effects of cognitive impairment and *APOE* genotype on the left insula (LIns) and right insula (RIns) networks across all subjects, voxel‐wise 4 × 2 ANCOVA (group × *APOE* genotype) was performed after controlling for covariates including gender and gray matter volume (GMV). The thresholds were set at *p* < 0.001 at individual voxels and corrected cluster *α* < 0.05 (cluster size ≥30 voxels) which was determined by Monte Carlo simulation for multiple comparisons after being corrected with the new version of 3dClustSim program (https://afni.nimh.nih.gov/pub/dist/doc/program_help/3dClustSim.htm).

#### 2.2.3 Phenotypic regression analyses

To investigate the correlations between CSF biomarkers (Aβ, Tau, and pTau), altered IFCs (the brain regions from main effects and interaction of the *APOE* genotype and disease status) and cognitive performance across the AD spectrum. Linear and binomial nonlinear regression analyses were employed after controlling for covariates of age, sex, education, *APOE* ε4 status, and GMV in subjects with CSF biomarkers.

#### 2.2.4 Mediation and moderation analysis

Given that significant linear correlation between CSF Tau, IFC, and cognitive performance across the AD spectrum (Figure 3) and the reported association between *APOE* genotype and cognitive function,^18^ mediation and moderation analysis were performed to further examine whether LIns network connectivity could moderate the effects of the *APOE* genotype and CSF Tau on cognitive performance across the AD spectrum. Herein, our mediation and moderation analysis have two purposes: (a) to test whether specific IFC (mediator) mediates the relationship between *APOE* genotype, CSF Tau, and cognitive function (PROCESS model 4) and (b) to further test whether the IFC (moderator) moderates the relationship between *APOE* genotype, CSF Tau and cognitive function (PROCESS model 1), with simple slope analysis to find out when under what circumstances and in what magnitude such moderated effect exists. The Johnson‐Neyman (JN) technique also allows the exact computation of conditions and boundary values where a moderator elicits statistically significant slopes. The bootstrapping method was applied to examine the significance of all the effects. The effects were significant if 95% bias‐corrected confidence intervals (CI) from bootstrapped analyses (5000 resamples) did not contain zero.

#### 2.2.5 Classification analysis

*APOE* genotype, CSF biomarkers, insula volumes, and altered IFCs were employed as distinguishing features to input the support vector classifier (SVC). Briefly, we used support vector machine (SVM) package in the LIBSVM toolbox to obtain optimal classifiers and test the power of classification.^19^ Due to the current limited sample size, the leave‐one‐out cross‐validation (LOOCV) method was used to estimate model performance. The accuracy was defined as the proportion of samples correctly classified. The performance of the classifier was estimated with receiver operating characteristic curves (ROC) by calculating the area under the curve (AUC). The AUC of the ROC curve is thus often considered the most useful global marker of the diagnostic accuracy. Detailed information can be found in the Appendix S1.

## 3 RESULTS

### 3.1 Demographic information and neuropsychological data

No significant differences were found in age, education or gender among all the participants (all *p* > 0.05). The AD group had smaller GM volumes compared to CN, SMC and MCI subjects. As the disease progressed, there was a gradually declining trend in MMSE, RAVLT‐I, RAVLT‐L and CSF Aβ and an increasing trend in ADAS‐cog, CSF Tau and CSF pTau. The *APOE* ε4 carriers represent higher CSF Tau and CSF pTau and lower CSF Aβ than that of *APOE* ε4 non‐carriers. More details are shown in Table 1.

**TABLE 1:** Demographic information and neuropsychological performance.

	CN		SMC		MCI		AD		*p*‐value		
	ε4+ (*n* = 14)	ε4− (*n* = 32)	ε4+ (*n* = 22)	ε4− (*n* = 13)	ε4+ (*n* = 17)	ε4− (*n* = 24)	ε4+ (*n* = 22)	ε4− (*n* = 10)	Disease	Gene	Disease × gene interaction
Age (years)	71.81 ± 4.13	75.22 ± 6.29	73.37 ± 6.10	72.98 ± 5.97	70.91 ± 7.37	71.91 ± 8.78	74.65 ± 6.21	70.91 ± 8.59	0.42	0.68	0.29
Education (years)	16.57 ± 2.44	15.94 ± 2.18	17.14 ± 2.21	16.00 ± 3.39	17.82 ± 2.13	15.92 ± 2.83	15.45 ± 2.89	16.40 ± 2.59	0.18	0.16	0.22
Gender (F/M)	6/8	14/18	10/12	6/7	9/8	15/9	10/12	7/3			0.47
GMVs (mL)	574.60 ± 50.76	572.60 ± 46.77	577.58 ± 43.47	574.61 ± 52.42	553.76 ± 50.48	569.05 ± 36.16	516.34 ± 69.11	528.83 ± 80.28	<0.001^bde^	0.06	0.51
MMSE	28.43 ± 1.70	28.88 ± 1.16	28.86 ± 1.58	28.69 ± 1.49	27.53 ± 1.87	27.75 ± 1.48	22.36 ± 2.30	22.90 ± 2.77	<0.001^abcde^	0.25	0.75
ADAS‐cog	8.83 ± 3.06	9.48 ± 4.15	11.65 ± 5.82	9.70 ± 5.09	18.82 ± 6.63	15.60 ± 6.90	32.37 ± 8.34	33.38 ± 9.10	<0.001^abcde^	0.08	0.69
RAVLT‐F	4.07 ± 2.43	3.97 ± 2.51	4.00 ± 2.64	3.85 ± 2.12	5.35 ± 1.69	4.08 ± 2.30	4.05 ± 1.21	5.30 ± 2.26	0.77	0.23	0.03
RAVLT‐I	41.00 ± 5.43	45.25 ± 11.26	43.59 ± 12.08	43.54 ± 7.61	34.41 ± 6.60	32.25 ± 8.08	20.82 ± 6.29	24.40 ± 8.28	<0.001^abcde^	0.60	0.41
RAVLT‐L	6.00 ± 2.51	5.38 ± 2.27	5.77 ± 2.45	6.23 ± 2.77	2.35 ± 1.41	4.25 ± 2.56	1.32 ± 1.86	2.90 ± 2.23	<0.001^abcde^	0.06	0.13
CSF Aβ (pg/mL)	190.92 ± 70.10	188.62 ± 44.75	175.25 ± 45.37	220.82 ± 41.76	142.87 ± 37.66	188.43 ± 52.14	129.60 ± 20.31	171.00 ± 61.63	0.001^bde^	0.001	0.02
CSF Tau (pg/mL)	69.41 ± 33.82	62.98 ± 35.67	82.70 ± 33.58	63.44 ± 24.58	109.59 ± 57.06	78.85 ± 50.94	140.67 ± 78.40	100.14 ± 38.83	0.003^abde^	0.001	0.54
CSF pTau (pg/mL)	42.19 ± 20.91	31.61 ± 14.71	49.50 ± 26.02	27.19 ± 6.21	52.49 ± 25.76	40.68 ± 21.06	55.94 ± 27.27	52.23 ± 15.55	0.029^abd^	<0.001	0.28

*Note*: Significant differences were found in MMSE, ADAS‐Cog among all groups. *p* values were obtained by 2 × 4 ANCOVA (with *APOE* genotype and disease status) analysis except for gender (χ^2^ test). a‐f: post hoc analysis (Bonferroni correction) further revealed the source of ANCOVA difference (a: CN vs. MCI; b: CN vs. AD; c: SMC vs. MCI; d: SMC vs. AD; e: MCI vs. AD).

Abbreviations: AD, Alzheimer's disease; ADAS‐cog, 13‐item Alzheimer's Disease Assessment Scale‐cognitive subscale; Aβ, amyloid‐β 1–42; CN, cognitive normal; CSF, cerebrospinal fluid; F/M, female/male; GMVs, gray matter volumes; MCI, mild cognitive impairment; mL, milliliter; MMSE, mini‐mental state examination; pTau, phosphorylated tau; RAVLT‐F, Rey auditory verbal learning test‐forgetting; RAVLT‐I, Rey auditory verbal learning test‐immediate; RAVLT‐L, Rey auditory verbal learning test‐learning; SMC, subjective memory complain; Tau, total tau.

### 3.2 Insula volume

**FIGURE 1:**
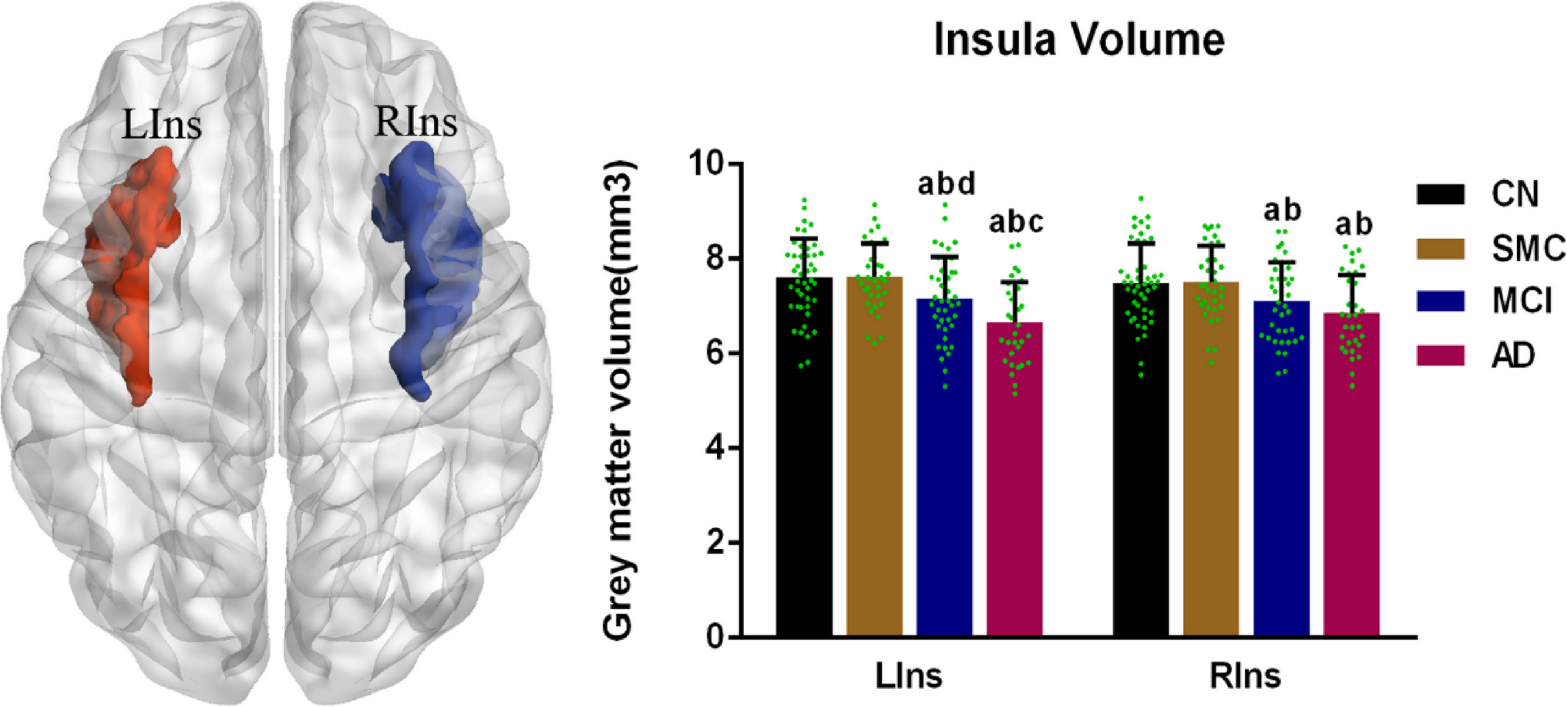
Insula volume assessment across AD spectrum. The bilateral insula, isolated using automated anatomical labeling, was interpolated to individual gray matter images. A mean volume index of all voxels of the insula region was computed for each subject. The bilateral insula volumes of MCI and AD subjects were smaller than those of CN, and SMC subjects (all *p* < 0.05). Moreover, the left insula volume of AD subjects was smaller than that of MCI subjects (*p* < 0.05). Each dot represented the insula gray matter volume of each participant. a–d: post hoc analysis further revealed the source of ANOVA difference (a: vs. CN; b: vs. SMC; c: vs. MCI, d: vs. AD). AD, Alzheimer's disease; CN, cognitive normal; LIns, left insula; MCI, mild cognitive impairment; RIns, right insula; SMC, subjective memory complaints.

The mean volume index of the insula region in each group was calculated by interpolating the insula to the individual gray matter images segmented from the T1 images. As presented in Figure 1, the volumes of the bilateral insula in the MCI and AD groups were significantly smaller than those in CN and SMC groups. Moreover, the AD group displayed smaller left insula volume than that of the MCI group (left, 7.68 ± 0.80 cm^3^ in CN group, 7.56 ± 0.72 cm^3^ in SMC group, 7.16 ± 0.87 cm^3^ in MCI group and 6.66 ± 0.86 cm^3^ in AD group; right, 7.75 ± 0.81 cm^3^ in CN group, 7.59 ± 0.76 cm^3^ in SMC group, 7.11 ± 0.80 cm^3^ in MCI group and 6.85 ± 0.81 cm^3^ in AD group).

### 3.3 Main and interactive effects of *APOE* genotype and disease status on the bilateral insula network

**FIGURE 2:**
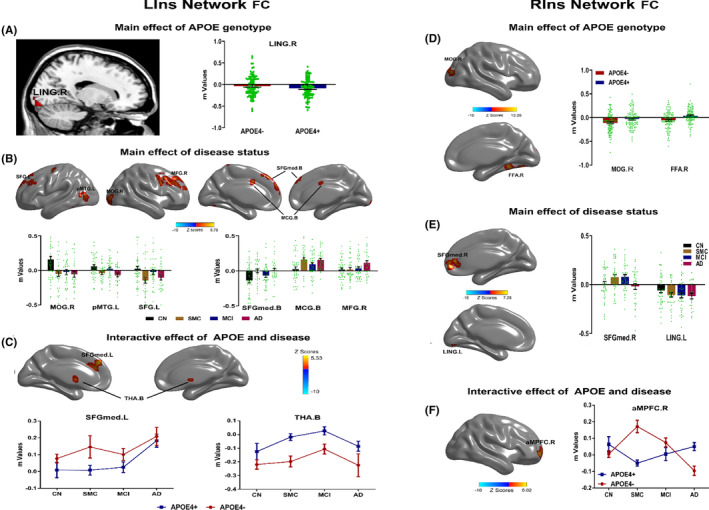
Main and interactive effects of the *APOE* genotype and disease status on the bilateral insula network across all participants. Brain regions significantly affected by *APOE* genotype were shown in (A) (LIns network) and (D) (RIns network). Brain regions with the main effects of disease status were shown in (B) (LIns network) and (E) (RIns network). The numerical representations of the significant main effects of the *APOE* genotype and disease status on bilateral insula networks were described using bar charts. Each dot represented the mean FC of each participant within significant brain regions in (A, B, D, E). During the progressive stages of the AD spectrum, from the SMC to AD stages, the distribution of the insula FC presented a disturbed pattern with U‐shaped and inverted U‐shaped trajectories. Brain regions with interactive effects of the *APOE* genotype and disease status were shown in (C) (LIns network) and (F) (RIns network). The numerical representations of the significant interactive effects of the *APOE* genotype and disease status on bilateral insula networks were illustrated with line charts. Importantly, the trajectory changes of RIns FC strength were opposite in *APOE* ε4 carriers (*APOE* ε4+) compared to *APOE* ε4 non‐carriers (*APOE* ε4–) across the AD spectrum. AD, Alzheimer's disease; aMPFC.R, right anterior medial prefrontal cortex; *APOE*, apolipoprotein E; CN, cognitive normal; FFA.R, right fusiform areas; LING.L, left lingual gyrus; LING.R, right lingual gyrus; LIns, left insula; MCG.L, middle cingulate gyrus; MCI, mild cognitive impairment; MFG.R, middle frontal gyrus; MOG.R, right middle occipital gyrus; pMTG.L, left posterior middle temporal gyrus; RIns, right insula; SFG.L, left superior frontal gyrus; SFGmed.B, bilateral medial superior frontal gyrus; SFGmed.L, left medial superior frontal gyrus; SFGmed.R, right medial superior frontal gyrus; SMC, subjective memory complaints; THA.B, bilateral thalamus.

The main effect of the *APOE* genotype on the insula network was primarily located in the right lingual gyrus (LING.R), right middle occipital gyrus (MOG.R), right fusiform areas (FFA.R) as described in Figure 2A,D. Compared with *APOE* ε4 non‐carriers, *APOE* ε4 carriers showed significantly higher intrinsic FC in LING.R and FFA.R while lower FC in MOG.R. The main effect of the disease was widely observed in the frontal‐temporal‐occipital system including the right middle occipital gyrus (MOG.R), left posterior middle temporal gyrus (pMTG.L), left superior frontal gyrus (SFG.L), bilateral medial superior frontal gyrus (SFGmed.B), bilateral middle cingulate gyrus (MCG.B), right middle frontal gyrus (MFG.R), right medial superior frontal gyrus (SFGmed.R), and left lingual gyrus (LING.L) as shown in Figure 2B,E. Intriguingly, the distribution of the insula network FC showed a disturbed pattern with dynamic changes along the AD process, especially from the SMC to AD stages. The pattern in MOG.R, pMTG.L, SFG.L, and SFGmed.R seemed to present an inverted U‐shape, whereas the pattern in the SFGmed.B and MCG.B showed a U‐shape. In addition, the FC strength in the MFG.R and LING.L increased throughout the course of AD. The interactive effect of the *APOE* genotype × disease on the insula network was found in the left medial superior frontal gyrus (SFGmed.L), bilateral thalamus (THA.B), and right anterior medial prefrontal cortex (aMPFC.R), as seen in Figure 2C,F. Compared to *APOE* ε4 non‐carriers, the *APOE* ε4 carriers produced opposite trajectory changes in aMPFC.R throughout the course of AD, also from the SMC to MCI stage in SFGmed.L. In addition, subjects with or without *APOE* ε4 represented increased connectivity from the CN to MCI stages and decreased connectivity from the MCI to AD stages as displayed in THA.B. For details of all the above‐mentioned brain regions, please refer to Tables S1 and S2.

### 3.4 Behavioral significance

**FIGURE 3:**
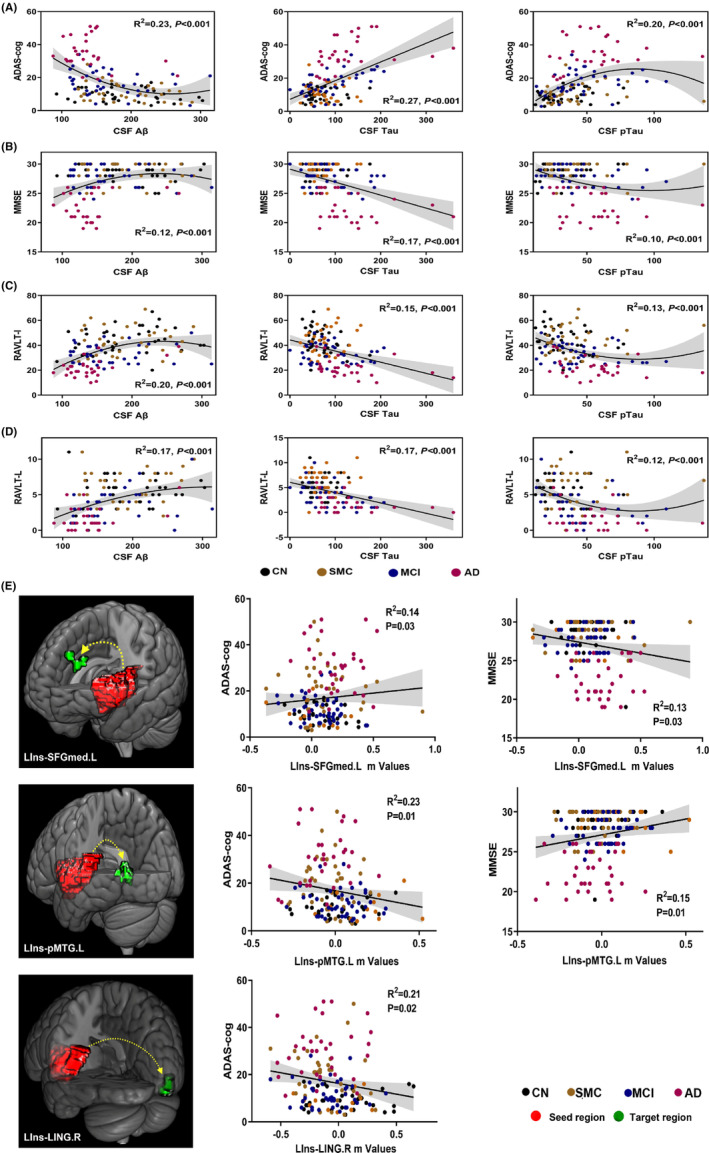
Regression analyses between CSF biomarkers, altered insula FCs, and cognitive performance across the AD spectrum. (A–D) Correlations between CSF biomarkers and cognitive performance across the AD spectrum. Nonlinear correlations were found between CSF Aβ, CSF pTau, and cognitive performance while linear correlation existed between CSF Tau and cognitive performance. (E) Linear correlations between altered insula FC and cognitive performance across the AD spectrum. AD, Alzheimer's disease; ADAS‐cog, 13‐item Alzheimer's Disease Assessment Scale‐Cognitive subscale; Aβ, amyloid‐β 1–42; CN, cognitive normal; CSF, cerebrospinal fluid; FC, functional connectivity; LING.R, right lingual gyrus; LIns, left insula; MCI, mild cognitive impairment; MMSE, Mini‐Mental State Examination; pMTG.L, left posterior middle temporal gyrus; pTau, phosphorylated tau; RAVLT‐I, Rey auditory verbal learning test‐immediate; RAVLT‐L, Rey auditory verbal learning test‐learning; RIns, right insula; SFGmed.L, left medial superior frontal gyrus; SMC, subjective memory complaints; Tau, total tau.

As shown in Figure 3A–D, the nonlinear regression analysis displayed that the CSF biomarkers Aβ and pTau were correlated with global cognitive scale scores (MMSE and ADAS‐cog) and episodic memory scale scores (RAVLT‐I and RAVLT‐L), while CSF Tau was linearly correlated. It is noted that the correlations were not significant between the CSF biomarkers and other cognitive scales (Figure S1). Regression analysis also disclosed that LIns‐SFGmed.L and LIns‐pMTG.L FCs could significantly predict MMSE and ADAS‐cog scores in a linear manner. LIns‐LING.R FC was also associated with ADAS‐cog but not with MMSE (Figure 3E, Figure S2).

### 3.5 Relationship between *APOE* genotype, CSF Tau, IFC and cognitive performance

**FIGURE 4:**
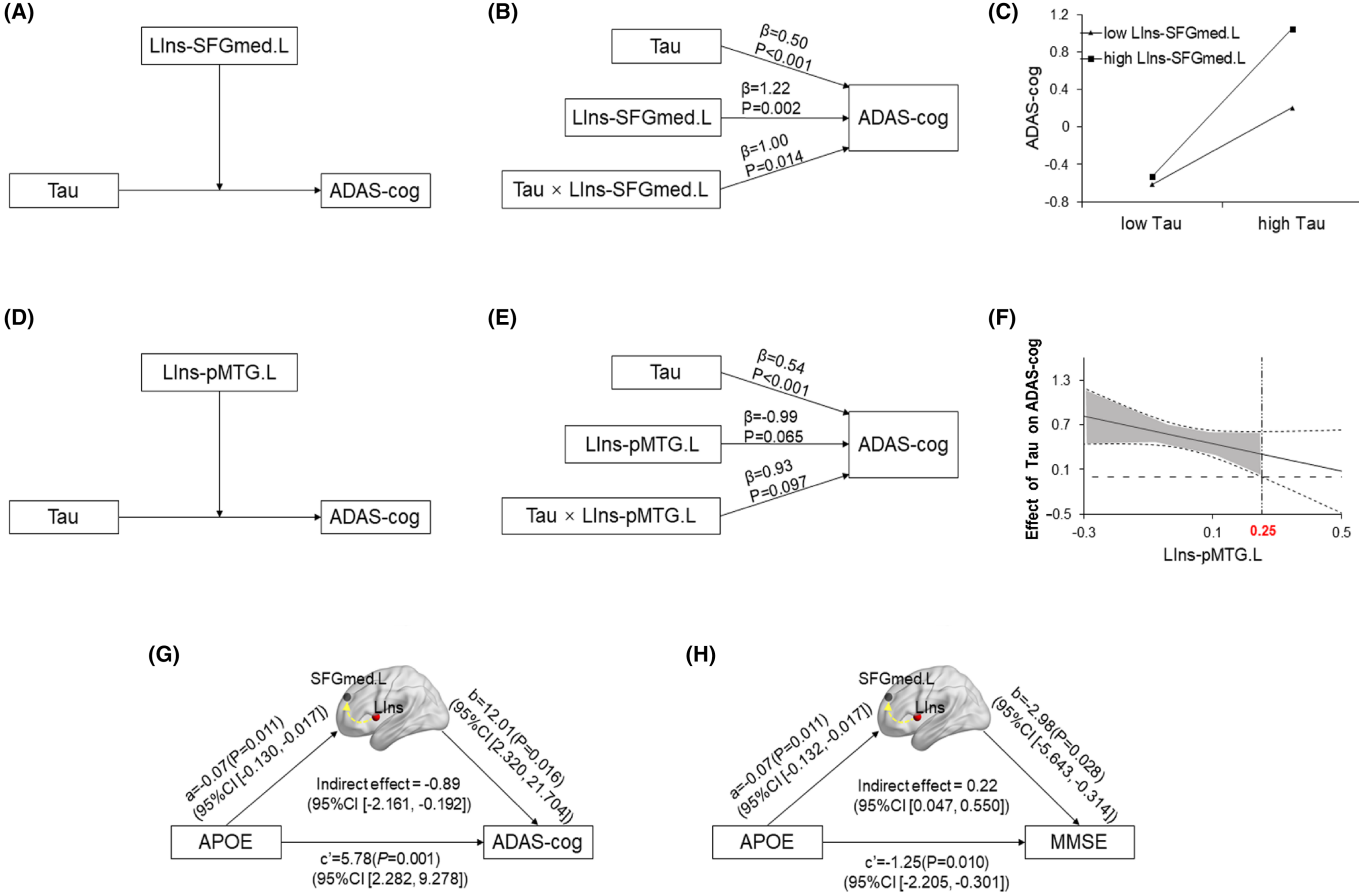
Moderation and mediation analysis. (A, D) The proposed moderation model. (B, E) Statistical diagrams present the effects of CSF Tau (*β* = 0.50, *p* < 0.001), LIns‐SFGmed.L (or LIns‐pMTG.L) connectivity (*β* = 1.22, *p* = 0.002, *β* = −0.99, *p* = 0.065, respectively), and interaction of CSF Tau with LIns‐SFGmed.L (or LIns‐pMTG.L) connectivity (*β* = 1.00, *p* = 0.014, *β* = 0.93, *p* = 0.097, respectively) on ADAS‐cog across the AD spectrum. (C) ADAS‐cog scores varied according to the interaction of CSF Tau and LIns‐SFGmed.L connectivity. The Slope diagram of CSF Tau against ADAS‐cog was graphed at both levels of the LIns‐SFGmed.L connectivity; one standard deviation above the mean and one standard deviation below the mean. All inferential analyses maintained the continuous values of CSF Tau and the strength of LIns‐SFGmed.L. (F) Johnson‐Neyman method was applied to perform a simple slope test. The black continuous lines showed the conditional effects of CSF Tau on ADAS‐cog depending on the LIns‐pMTG.L FC values. In addition, the dotted lines above and below indicate the corresponding 95% confidence intervals (CI). (G, H) The proposed mediation model. Mediation analysis revealed that the LIns‐SFGmed.L connectivity mediated the relationship between the *APOE* genotype and ADAS‐cog (or MMSE) across the AD spectrum. ADAS‐cog, 13‐item Alzheimer's Disease Assessment Scale‐Cognitive subscale; Aβ, amyloid‐β 1–42; CSF, cerebrospinal fluid; FC, functional connectivity; LIns, left insula; MMSE, Mini‐Mental State Examination; pMTG.L, left posterior middle temporal gyrus; SFGmed.L, left medial superior frontal gyrus; Tau, total tau.

The moderation process analysis revealed that LIns‐SFGmed.L and LIns‐pMTG.L FCs, not LIns‐LING.R connectivity significantly moderated the causal effect of CSF Tau on the ADAS‐cog across all groups (Figure 4A–F, Figure S3). The relationship between two variables (CSF Tau and ADAS‐cog) was moderated when its size or sign depends on the third variable (LIns‐SFGmed.L and LIns‐pMTG.L FCs). To begin with, as shown in Figure 4A,B, the moderation model indicated that there was a significant main effect of CSF Tau on ADAS‐cog (*β* = 0.50, *p* < 0.001), and more importantly, this effect was moderated by LIns‐SFGmed.L FC (*β* = 1.00, *p* = 0.014). For descriptive purposes, the study plotted predicted ADAS‐cog scores against CSF Tau, separately for low and high levels LIns‐SFGmed.L FC (one SD below the mean and one SD above the mean, respectively) (Figure 4C). It appears that the effect of LIns‐SFGmed.L FC on cognitive function is larger among those with higher CSF Tau, represented by the growing gap between the two lines with increasing CSF Tau. As can be seen, the higher LIns‐SFGmed.L FC, the worse cognitive function. Simple slope tests demonstrated that for subjects with high levels of LIns‐SFGmed.L FC, higher levels of CSF Tau were associated with higher ADAS‐cog scores, *b*_simple_ = 0.77, *p* < 0.001. However, for the subject with low levels of LIns‐SFGmed.L FC, higher levels of CSF Tau were associated with lower ADAS‐cog scores compared to high LIns‐SFGmed.L FC carriers, *b*_simple_ = 0.41, *p* < 0.001. Figure 4F presented a significantly negative relationship of LIns‐pMTG.L moderating the effect of CSF Tau on the cognitive performance. That is, the CSF Tau produced a negative effect on cognitive performance with an increased LIns‐pMTG.L connectivity. Furthermore, this moderating effect significant only existed when the LIns‐pMTG.L value varied on the interval (−0.30, 0.25). That also means the higher LIns‐pMTG.L FC, the better cognitive function. Notably, we did not find insula network connectivity could significantly moderate the relationship between *APOE* genotype and cognitive performance (Figure S5). Finally, as shown in Figure 4G,H LIns‐SFGmed.L FC significantly mediated the effects of *APOE* genotype and cognitive performance (ADAS‐cog and MMSE) across all groups. *APOE* had a positive effect on ADAS‐cog and negative effect on MMSE across all groups. Conversely, LIns‐SFGmed.L connectivity mediated the negative indirect effect of *APOE* genotype on ADAS‐cog and the positive indirect effect of *APOE* genotype on MMSE indicating that the relationship between *APOE* and cognition was suppressed by IFC. Namely, *APOE* ε4 carriers with higher LIns‐SFGmed.L FC might predict lower ADAS‐cog and higher MMSE. However, we did not find insula network connectivity could significantly mediate the relationship between CSF Tau and cognitive performance (Figure S4).

### 3.6 Classifying AD spectrum population with SVM model

**FIGURE 5:**
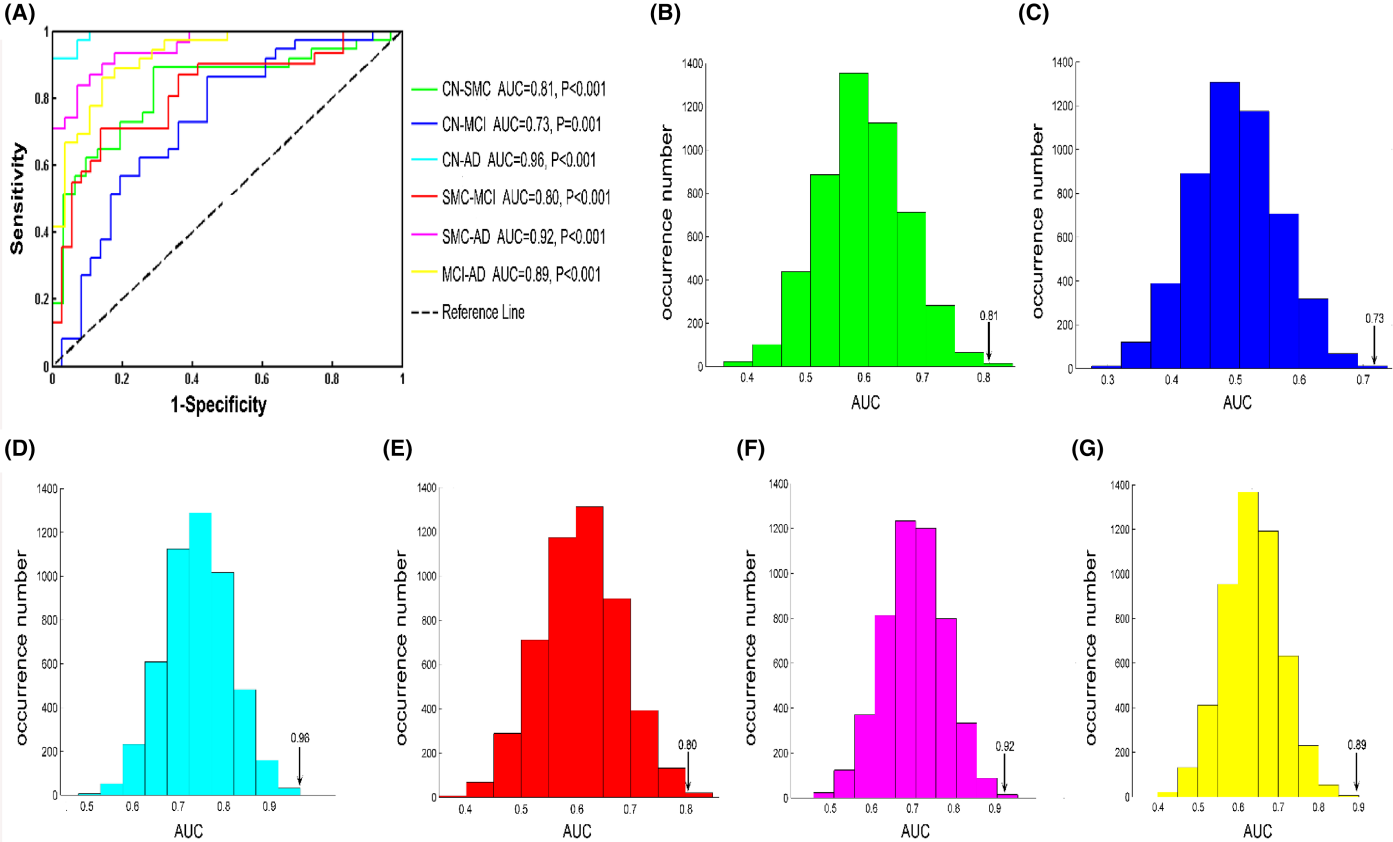
ROC curves from the support vector machine for classifying the AD spectrum. (A) SVM model integrated *APOE* genotype, cerebrospinal fluid biomarkers, and neuroimaging biomarkers produced strong power for classifying CN from SMC (AUC = 0.81), CN from MCI (AUC = 0.73), CN from AD (AUC = 0.96), SMC from MCI (AUC = 0.80), SMC from AD (AUC = 0.92) and MCI from AD (AUC = 0.89). (B–G) Permutation distribution of the AUCs (green represents the CN‐SMC, blue represents CN‐MCI, cyan represents CN‐AD, red represents SMC‐MCI, magenta represents SMC‐AD, yellow represents MCI‐AD) from the machine learning classification analysis. Permutation tests for all AUCs were statistically significant (*p* < 0.001). AD, Alzheimer's disease; AUC, area under curve; CN, cognitive normal; MCI, mild cognitive impairment; ROC, receiver operating characteristic; SMC, subjective memory complaints; SVM, support vector machine.

When we employed the SVM‐trained model as a classifier, which integrates the *APOE* genotype, CSF biomarkers (Aβ, Tau, and pTau), and neuroimaging biomarkers (insula volumes and altered regional IFCs based on the *APOE* genotype), The model produced a strong power for classifying different disease stages. The classification presented high accuracy, sensitivity, and specificity (all values >0.70, Table 2). As illustrated in Figure 5A, all AUC values were more than 0.72, indicating the good power of combining these predictive variables to discriminate disease stages. AUC values of the classifier which were cross‐validated by the permutation test were statistically significant (all *p* < 0.001) (Figure 5B–G).

**TABLE 2:** Capacities for classifying AD spectrum derived from the SVM.

	Accuracy	Sensitivity	Specificity
CN vs. SMC	0.74	0.73	0.78
CN vs. MCI	0.71	0.86	0.76
CN vs. AD	0.94	0.92	0.96
SMC vs. MCI	0.72	0.71	0.86
SMC vs. AD	0.88	0.84	0.93
MCI vs. AD	0.84	0.86	0.85

Abbreviations: AD, Alzheimer's disease; CN, cognitive normal; MCI, mild cognitive impairment; SMC, subjective memory complaints; SVM, support vector machine.

## 4 DISCUSSION

In this study, we demonstrated the main and interaction of the *APOE* genotype and disease status on the insula network within the frontal‐temporal‐occipital system in the *APOE* ε4 carriers compared to non‐carriers across the AD spectrum. Additionally, CSF biomarkers including Aβ and pTau could impact cognition in a nonlinear manner while Tau in a linear manner. We also found LIns‐SFGmed.L connectivity could mediate the effects of *APOE* genotype on cognition. Furthermore, LIns‐SFGmed.L and LIns‐pMTG.L connectivities could moderate the effects of CSF Tau on cognition. More importantly, the integration of *APOE* genotype, CSF biomarkers, and neuroimaging biomarkers into the trained SVM model yielded strong powers to classify the AD spectrum. These findings indicated the insula network connectivity moderated the effects of the *APOE* genotype, CSF Tau on cognition and provided stage‐dependent biomarkers for early differentiation of the AD spectrum.

A longitudinal follow‐up study has shown that insula atrophy is significant in progressive MCI.^20^ In the present study, we found bilateral insula atrophy in MCI compared to CN and SMC and further left insula atrophy but no right insula atrophy in AD compared to MCI. The main effect of the *APOE* genotype on the insula network focused on LING.R, MOG.R, and FFA.R. These abnormal connectivities of the visual–auditory pathway might alter the semantic memory network.^21^ The insula network affected by the disease status is primarily located in the frontal‐temporal‐occipital system and was frequently reported damaged areas in the progression of AD, preferentially associated with Tau deposition.^17^, ^22^, ^23^ Structurally, there are projections between the insula and frontal‐temporal‐occipital regions, and these regions are functionally involved in perception, attentional processing and cognitive control processes.^24^, ^25^, ^26^ The distributions of altered IFCs in the MOG.R, pMTG.L, SFG.L, and SFGmed.R were more likely to be inverted U‐shape, suggesting that the FCs strength increased from the SMC to MCI stage but decreased to the AD stage. These changing FC strengths might be interpreted as compensatory in MCI and disruption in AD.^27^, ^28^ On the contrary, the pattern in the SFGmed.B and MCG.B showed a U‐shape. In addition, the FC strength in the MFG.R and LING.L represented an increased trend in the progression of AD. Both cognitive ability and DMN FC decline in aging and are associated with the severity and progression of AD.^29^ DMN cortical hubs showed a large amount of Aβ deposition in AD, which might be due to the enhanced activity‐dependent processing of APP, as well as Tau hyperphosphorylation. The processing of information by older adults requires more resources. A model of the effects of aging on brain activity during cognitive performance is called HAROLD (hemispheric asymmetry reduction in older adults),^30^ in contrast to younger adults, older adults tend to have less lateralized prefrontal activity during cognitive performance under similar circumstances. Asymmetry reductions associated with aging may serve as a compensatory mechanism. The DMN is a set of widely distributed brain regions in the parietal, temporal, and frontal cortex. Our study validated the frontal compensation (e.g., Ins‐MFG FC) hypothesis across the AD spectrum. The dichotomous insula network pattern might be interpreted by different neural mechanisms underlying AD progression.^23^ An interactive effect between disease status and the *APOE* genotype was observed SFGmed.L, THA.B, and aMPFC.R. Significant IFC divergent trajectory across the AD spectrum in APOE ε4 carriers and non‐carriers were observed. Interestingly, the SFGmed.L and THA.B FCs in *APOE* ε4 carriers and non‐carriers showed a synergistic trend. SFGmed.L FC increased throughout AD progression while THA.B FC disrupted in the late stage of AD.^31^, ^32^ However, *APOE* ε4 carriers and non‐carriers showed approximately opposite aMPFC.R FC trajectories throughout AD progression, especially in the transition from SMC to MCI stages, which might be due to the modulating role of genetic profile in the predementia period.

Previous studies have shown that AD is associated with lower CSF Aβ and higher CSF Tau and pTau compared with elderly controls.^33^, ^34^ The study identified an increasing trend of pathological damage with the development of AD. The curving relationship between increased CSF biomarkers (Aβ and pTau) and declined cognition throughout the course of AD, whereas CSF Tau and cognitive performance were linearly correlated. In addition, we also demonstrated altered LIns‐SFGmed.L and LIns‐pMTG.L FCs could predict MMSE and ADAS‐cog scores across all the groups, respectively. LIns‐LING.R FC is also associated with ADAS‐cog but not with MMSE. SFGmed.L belongs to the frontal region and pMTG.L belongs to the temporal region. As neurotoxicity continued to expand during AD progression, researchers have found excessively strengthened intrinsic neuroactivities in the frontal and temporal regions.^35^, ^36^, ^37^, ^38^ Our result suggested that the more increased connectivity in the LIns‐pMTG.L and LIns‐LING.R, the better the cognitive function. As the FCs diminished, the severity of damage outweighed compensation, which leads to irreversible disruption of general cognition.

This study established mediation and moderation models to test whether *APOE* genotype and CSF Tau would also be indirectly associated with cognition through IFC. The relationship between *APOE* genotype and cognition in AD or MCI has been reported.^39^, ^40^, ^41^ Hippocampus atrophy in AD or MCI may mediate the effect of *APOE* ε4 on memory function.^42^ Amygdala FC strength could regulate the effects of the APOE genotype and age on cognitive function in aMCI patients.^43^ Our previous study found that DMN connectivity might modulate the association between *APOE* genotype and cognition across AD spectrum population.^11^ The current mediation results indicated that the effect of the *APOE* genotype on cognition could be partially explained by LIns‐SFGmed.L FC. Specifically, the *APOE* genotype would negatively predict LIns‐SFGmed.L FC, and in turn, LIns‐SFGmed.L FC could positively predict ADAS‐cog and negatively predict MMSE. In other words, *APOE* ε4 showed significant direct harmful effect and indirect protective effect on cognitive function through LIns‐SFGmed.L FC. It suggested that LIns‐SFGmed.L FC carried a suppression effect in the link between *APOE* genotype and cognition. Several studies have shown that the SFGmed plays an important regulatory role in cognitive functions, including attention, inhibitory control and working, spatial or long‐term memory.^44^ The SFGmed interconnects with subcortical areas (thalamus, amygdala, and hippocampus) and exerts top‐down executive control over a variety of cognitive domains and stimuli.^45^, ^46^ Previous studies identified four distinct spatiotemporal trajectories of Tau pathology.^47^ Pathology originates and spreads through distinct corticolimbic networks in the different subtypes. Our findings (mainly CSF Tau) complement AD studies from the Tau pathology spatial pattern. Recently, Hsu et al. reported that structural MRI (diffusion tensor image analysis along the perivascular space, ALPS‐index) acted as a significant mediator between Tau and cognitive dysfunction in AD patients. These regions are responsible for attention, memory, and executive function, which are vulnerable to sleep deprivation.^48^ In early‐onset AD and late‐onset AD subjects, higher CSF Tau values at baseline predicted higher rates of subcortical atrophy and might be a relevant molecular biomarker that could indicate disease pathology and/or progression in AD, which was often accompanied by axonal degeneration.^49^, ^50^ The current moderation results suggested significant interaction between CSF Tau and IFC on cognitive function. Tau pathology initially accumulates in the entorhinal cortex/hippocampus and may spread prion‐like Tau from the MTG to the SFG.^51^ Combined with our result, CSF Tau could positively predict ADAS‐cog. And this direct effect was moderated by LIns‐SFGmed.L and LIns‐pMTG.L FCs (*β* = 1.00, *β* = 0.93, respectively). On the one hand, High LIns‐SFGmed.L FC and high level of CSF Tau lead to worse cognition compared to low LIns‐SFGmed.L FC and low level of CSF Tau among all the subjects. This is consistent with the interaction effect above, the AD group has the highest LIns‐SFGmed.L FC, highest level of CSF Tau, and the worst cognition compared to other groups. On the other hand, LIns‐pMTG.L FC moderated the relationship between CSF Tau and cognition. The higher LIns‐pMTG.L FC, the weaker effect of CSF Tau on cognition. In other words, subjects with higher LIns‐pMTG.L FC can reduce the harmful effect of CSF Tau on cognitive function. Furthermore, we identified this significant effect only existed when the LIns‐pMTG.L FC value varied on the interval (−0.30, 0.25) using the Johnson‐Neyman technique. A typical feature of SMC is hypometabolism of MTG, while large‐scale hypometabolism of AD starts from MTG and develops gradually from the preclinical stage.^52^ MTG is also an important component of DMN, with close functional links to the hippocampus, and is mainly involved in verbal or semantic cognition and also associated with oral memory.^53^ in addition, it is a hallmark area of cortical atrophy in AD patients.^54^, ^55^ Lim et al.^56^ showed that the MTG atrophied as early as the SMC stage. These results provide a new perspective on the mechanisms that promote the interaction between the genetic risk factor, CSF Tau, and cognitive performance. To our best knowledge, the current findings furnish the first evidence that insula connectivity has a dual role, mediating the link between *APOE* genotype and cognition, and moderating the relationship between CSF Tau and cognition. Taken together, these findings strongly suggested that IFC might bridge the bridge between CSF Tau, *APOE* genotypes, and cognitive function, constructing a gene‐brain‐behavior path across the AD spectrum.

Interconnection patterns of entire brain regions have been reported to be useful in classifying subjects with AD, MCI, and CN in large‐scale network analysis.^57^ Brain atrophy quantification and ADAS‐cog have similar values in predicting the progression of MCI to AD.^58^ Their combination was superior than their individual alone.^59^ Positron emission tomography (PET) imaging of amyloid has radiation risks and is expensive.^60^, ^61^ Cognitive scale evaluation has subjective factors. Given these facts, we used SVM which integrated *APOE* genotype, CSF biomarkers (Aβ, Tau, and pTau), and neuroimaging biomarkers (insula volumes and altered regional IFC strengths) to classify AD spectrum population who are difficult to assess or stage diagnosis. The classifier achieved high accuracy, stable sensitivity, and specificity which displayed good ability to distinguish between any two groups. Notably, the classifier could not distinguish the five groups at one time. The result was also verified using the leave‐one‐out cross‐validation analysis.

To date, we first reported that the IFC conditionally regulates the association between CSF Tau and cognition. This implies that we can use non‐invasive neuroregulatory techniques (e.g., transcranial magnetic stimulation therapy) to intervene IFC to treat AD's cognition. For AD patients, a precision medicine strategy is needed to address the heterogeneous pathophysiology of AD and its diverse symptoms.^62^ Some recent studies have found single‐molecule polypharmacology that could multi‐target the disease pathogenesis.^63^, ^64^, ^65^, ^66^ Based on our results, the precision medical strategy includes collecting demographic information (e.g., APOE genotype, age, gender, education) of each patient as the basis for AD patient stratification, and incorporating in‐depth information to promote therapies, such as physical activity, diet, brain stimulation (insula functional network‐focused therapies) and drug therapy. This study had several limitations. First, the results of this study should be taken with caution. Mediated and moderated models contain causal paths that imply the *APOE* (or CSF Tau)‐behavior, and testing these paths with cross‐sectional data can produce biased estimates.^67^ When possible, and absent the possibility of implementing an experimental design,^68^ future research should assess mediation and moderation using longitudinal data, preferably with panel models that allow the comparison of alternative causal flows. Second, this study was conducted in a small sample of AD spectrum subjects with *APOE* genotype and CSF biomarkers, so more subjects should be enrolled to validate our results. Third, we found that the SVM model integrating *APOE* genotype, CSF biomarkers, and neuroimaging biomarkers had good power for the classification of the AD spectrum. Although leave‐one‐out cross‐validation analysis was used, future studies will need to be conducted on a new dataset to validate the present results.

## 5 CONCLUSION

We demonstrated that the *APOE* gene could affect the insula network across the AD spectrum population. The moderation and mediation analysis revealed that intrinsic IFC could regulate the effects of CSF Tau and *APOE* gene on cognition. Finally, we propose that the combination of the *APOE* genotype, CSF biomarkers, and altered IFCs may serve as stage‐dependent biomarkers for differentiation of the AD spectrum. These findings deepen our understanding of the relationship between *APOE* gene, CSF Tau, insula network connectivity, and cognition and provide novel insights into objective diagnostic biomarkers for AD spectrum classification.

## CONFLICT OF INTEREST STATEMENT

The authors declare no conflicts of interest.

## CONSENT

Written informed consent was obtained from all participants and authorized representatives, and the study partners before any protocol‐specific procedures were carried out in the ADNI study. More details in http://www.adni‐info.org.

## Supporting information


Appendix S1

## Data Availability

The data that support the findings of this study are openly available in ADNI database at http://adni.loni.usc.edu.
