# Supplementary material for: The relationship between APOE genotype, CSF Tau and cognition across the Alzheimer's disease spectrum, moderation and mediation role of insula network connectivity
Source: CNS Neurosci Ther. 2023 Aug 14;30(1):e14401. doi: 10.1111/cns.14401 (PMC10805399; doi:10.1111/cns.14401)
Supplement: Supplementary file 1 — Appendix S1 [file CNS-30-e14401-s001.docx]

Supplementary Material

**Title:** **The relationship between APOE genotype,** **CSF Tau and cognition across the Alzheimer’s Disease Spectrum, moderation and mediation role of** **insula network connectivity**

Yao Zhu^1^*, Yan Wu^1^, Xinyi lv^1^, Jiaonan Wu^1^, Chunzi Shen^1^, Qiqiang Tang^1^*, Guoping Wang^1^* on behalf of Alzheimer’s disease Neuroimaging Initiative

^1^Department of Neurology, The First Affiliated Hospital of USTC, Division of Life Sciences and Medicine, University of Science and Technology of China, Hefei, China, 230001

This supplemental material include:

Materials and methods

Neuropsychological assessment

Statistical analysis

Table S1-S2

Figure S1-S5

**Materials and methods**

**Alzheimer’s Disease Neuroimaging Initiative (ADNI) database**

The ADNI was launched in 2003 as a public private partnership, led by Principal Investigator Michael W. Weiner, MD. The primary goal of the ADNI study is to track the progression of the disease using biomarkers, together with clinical measures, to assess the brain’s structure and function over the course of disease states. The ADNI was comprised of initial 5-year ADNI1 study, extended 2-year ADNIGO study, and further competitive renewals in the year of 2011 and 2016 (ADNI2 and ADNI3). This is a non-randomized natural history non-treatment study to determine the relationships among clinical, imaging, and genetic characteristics of the entire spectrum of AD. The goal of ADNI neuropsychological testing is to use standardized procedures to assess a subject’s cognitive abilities objectively and reliably. All subjects will have clinical/cognitive assessments and MRI at specified intervals (6 or 12 month) for 2-3 years. The sequence of cognitive tests is as follows: Mini Mental State Examination, Logical Memory I and II, Auditory Verbal Learning Test, Alzheimer’s Disease Assessment Scale-Cognitive (ADAS), Clinical Dementia Rating Scale. More information could be found at <https://adni.loni.usc.edu/methods/documents/>.

**Informed Consent**

Written informed consent was obtained from all participants, authorized representatives, and study partners before any protocol-specific procedures were carried out in the ADNI study.

**Inclusion Criteria**

The diagnostic criteria were described in the ADNI manual (http:// [www.adni-info.org](http://www.adni-info.org)). CN subjects had no memory complaints. SMC subjects had self-reported persistent memory decline and normal cognitive performance. The CN and SMC subjects had Mini-Mental State Examination (MMSE) score between 24 and 30 and clinical dementia rating (CDR) score of 0. MCI subjects had a subjective memory concern as reported by the subject, study partner or clinician. The MCI subjects’ MMSE scores were between 21 and 30, CDR scores of 0.5, objective memory loss measured through Logical Memory Test (Delayed Paragraph Recall) (=8 for 16 years and more of education; =4 for 8–15 years of education; and =2 for 0–7 years of education). For mild AD subjects, the MMSE scores ranged from 20 to 26, a CDR equalled to 0.5 or 1.0 and met National Institute of Neurological and Communicative Diseases and Stroke/Alzheimer's Disease and Related Disorders Association (NINCDS/ADRDA) criteria for probable AD.

**CSF biomarkers**

CSF sample preparation were performed as described in the ADNI manual (http://adni.loni.usc.edu/research/protocols/biospecimens-protocols/). CSF amyloid-β 1-42 (Aβ), total Tau (Tau) and phosphorylated tau (pTau) were measured using INNOBIA AlzBio3 immunoassay kit-based reagents (Innotest, Fujirebio, Ghent, Belgium). Since lumbar puncture is an invasive operation, not all subjects had CSF sample data. After exclusion of incomplete or missing information regarding CSF sample data, 37 out of 46 CN subjects, 31 out of 35 SMC subjects, 36 out of 41 MCI subjects and 28 out of 32 AD subjects were sorted out for final analysis.

**Data acquisition**

All subjects were scanned on a 3.0-Tesla Philips MRI scanner. rs-fMRI images were obtained using an echo-planar imaging sequence with the following parameters: 140 time points, repetition time (TR) = 3000 ms, echo time (TE) = 30 ms, flip angle = 80°, number of slices = 48, slice thickness = 3.3 mm, spatial resolution = 3×3×3 mm^3^, acquisition matrix = 64×64, and field of view (FOV) = 240 × 240 mm. All original image files were available to the general scientific community. T1-weighted images were acquired using a sagittal magnetization prepared rapid gradient echo (MP-RAGE), with data parameters: TR = 6700 ms, TE = 3.1 ms, slice thickness = 1.2 mm, FA = 9°,

FOV = 250 × 250 mm, thickness = 1.0 mm, gap = 0 mm, and number of slices = 170.

**Data preprocessing**

The data preprocessing was performed using the Data Processing & Analysis for Brain Imaging (DPABI V4.1, <http://rfmri.org/dpabi/>) and Statistical Parametric Mapping (SPM12, <http://www.fil.ion.ucl.ac.uk/spm>) in MATLAB 2012b(MathWorks, Inc; Natick, Massachusetts). Briefly, the first 10 volumes of the scanning session were discarded to allow for T1 equilibration effects. The remaining images were corrected for timing differences and motion effects. No translation or rotation parameters of head motion in any given data set exceeded ± 3 mm or ± 3°. The resulting images were spatially normalized to the standard Montreal Neurological Institute (MNI) echo-planar imaging template using the default settings, resampling to 3 × 3 × 3 mm^3^ voxels, and smoothed with a Gaussian kernel of 6 × 6 × 6 mm. To further reduce the effects of confounding factors, the Friston’s 24 head motion parameters, as well as white matter (WM) signal, and cerebrospinal fluid (CSF) signal, were regressed out. Finally, a bandpass filter was applied to keep only low-frequency fluctuations between 0.01-0.08 Hz.

**Voxel-wised insula** **functional connectivity analysis**

To create seeds for the functional connectivity (FC) analysis, bilateral insula regions were separately defined using the automated anatomical labelling implemented with WFU_PickAtlas software (Maldjian et al, 2003). The defined seed regions were then resampled to the same space as the functional data. For each subject, the averaged time series of the seed region was computed as the reference time course. Then, a Pearson cross-correlation analysis was performed between the seed time course and the time course of the all-brain voxels. Fisher's z-transformation was applied to improve the normality of the correlation coefficients (CC) [m = 0.5ln(1+CC)/(1-CC)]. In this way, individual insula network maps were obtained.

**Structural image analysis and insula volume assessment**

The gray matter volume (GMV) was considered as an covariate in the FC analysis(Zhu et al, 2019; Zhu et al, 2020). An optimized voxel-based morphometry (VBM) analysis was conducted using Computational Anatomy Toolbox (CAT12, http://www.neuro.uni-jena.de/cat/) to calculate the GMV in all subjects. In brief, the structural images were normalized to the Montreal Neurological Institute (MNI) template using an affine and nonlinear spatial normalization. The normalized images were segmented into gray matter, white matter and cerebrospinal fluid according to MNI prior probability maps. Then, Jacobian modulation was applied to the segmented gray matter image, which can be incorporated to compensate for the effect of spatial normalization. Finally, the extracted gray matter set was smoothed with 8-mm full width at half maximum Gaussian kernel. The final images were regressed out as covariate of no interest when calculating FC. Next, the insula regions were interpolated to the same dimension, sizes, and origins with individual images. A mean volume index of all the voxels of the insula region was computed for each subject. The insula volume was obtained by multiplying the mean volume index by the number of voxels and the size of each voxel (Bai et al, 2009; Zhu et al, 2020).

**References**

Maldjian JA, Laurienti PJ, Kraft RA, Burdette JH. An automated method for neuroanatomic and cytoarchitectonic atlas-based interrogation of fMRI data sets. NeuroImage. 2003;19(3):1233-1239.

Zhu Y, Gong L, He C, Wang Q, Ren Q, Xie C. Default Mode Network Connectivity Moderates the Relationship Between the APOE Genotype and Cognition and Individualizes Identification Across the Alzheimer's Disease Spectrum. Journal of Alzheimer's disease : JAD. 2019;70(3):843-860.

Zhu Y, Zang F, Liu X, et al. Endocytosis-pathway polygenic scores affects the hippocampal network connectivity and individualized identification across the high-risk of Alzheimer's disease. Brain imaging and behavior. 2020.

Bai F, Zhang Z, Watson DR, Yu H, Shi Y, Yuan Y. Abnormal white matter independent of hippocampal atrophy in amnestic type mild cognitive impairment. Neuroscience letters. 2009;462(2):147-151.

**Neuropsychological assessment**

**Mini-Mental State Exam (MMSE) (Folstein et al,1975)**

The scale evaluates orientation to place, orientation to time, registration (immediate repetition of three words), attention and concentration (serially subtracting seven beginning with 100), recall (recalling the previously repeated three words), language (naming, repetition, reading, writing, comprehension), and visual construction (copy two intersecting pentagons). The MMSE is scored as the number of correctly completed items with lower scores indicative of poorer performance and greater cognitive impairment. The total score ranges from 0 to 30 (perfect performance).

**NINCDS-ADRDA Alzheimer's Criteria (McKhann et al,1984)**

Probable Alzheimer's disease: Dementia has been established by clinical and neuropsychological examination. Cognitive impairments also must be progressive and be present in two or more areas of cognition. The onset of the deficits has been between the ages of 40 and 90 years and finally there must be an absence of other diseases capable of producing a dementia syndrome.

**Clinical Dementia Rating (CDR) (Berg et al,1988)**

The CDR describes five degrees of impairment in performance on each of 6 categories of cognitive functioning including memory, orientation, judgment, and problem solving, community affairs, home and hobbies, and personal care. The ratings of degree of impairment obtained on each of the 6 categories of function are synthesized into one global rating of dementia (ranging from 0 to 3), with more refined measure of change available by use of the Sum of Boxes. Reliability and validity have been established, as has high inter-rater reliability. This will be used as a global measure of severity of dementia.

**Alzheimer’s Disease Assessment Scale-Cognitive (ADAS-cog) (Rosen et al, 1988)**

The ADAS-COG is a structured scale that evaluates memory (word recall, word recognition), reasoning (following commands), language (naming, comprehension), orientation, ideational praxis (placing letter in envelope) and constructional praxis (copying geometric designs). Ratings of spoken language, language comprehension, word finding difficulty, and ability to remember test instructions are also obtained. The test is scored in terms of errors, with higher scores reflecting poorer performance. Scores can range from 0 (best) to 70 (worse).

**Logical Memory Test (LMT) (Wechsler,1987)**

The Logical Memory test that will be used is a modification of the episodic memory measure from the Wechsler Memory Scale-Revised (WMS-R) (D Wechsler, 1987). In this modified version, free recall of one short story (Story A) that consists of 25 bits of information will be elicited immediately after it is read aloud to the subject and again after a thirty-minute delay. The total bits of information from the story that are recalled immediately (maximum score = 25) and after the delay interval (maximum score = 25) are recorded. A retention or “savings” score can be computed by dividing the score achieved during delayed recall by the score achieved during immediate recall.

**The Rey Auditory Verbal Learning Test (RAVLT)** **(Rey, 1958)**

RAVLT evaluates a wide diversity of functions: short-term auditory-verbal memory, rate of learning, learning strategies, retroactive, and proactive interference, presence of confabulation of confusion in memory processes, retention of information, and differences between learning and retrieval. Participants are given a list of 15 unrelated words repeated over five different trials and are asked to repeat. Another list of 15 unrelated words are given and the client must again repeat the original list of 15 words and then again after 30 minutes. Approximately 10 to 15 minutes is required for the procedure (not including 30 min. interval).

**Statistical analysis**

**Classification with the Support Vector Machine**

The classification was conducted using linear support vector machine (SVM) to differentiate any two groups of subjects across the AD spectrum. In the implementation, we used a SVM package which was bult in the MATLAB, LIBSVM toolbox (Chang and Lin, 2011), to get optimal classifiers and test the power of classification. SVM classification is a form of supervised learning because training is accomplished by labeled samples (Burges, 1998). A binary label with -1 and 1 for any two groups. Two steps were conducted in the classification process: training and testing. During the training step, the SVM calculates a decision boundary that separates the samples in the input space using their class labels. Once the decision function is determined from the training set, it can be used to predict the class label of a new testing example.

Evidence suggests that SVM with linear kernel is an appropriate model to avoid over-fitting and to allow direct extraction of the feature weights when training samples are small but the features number are large (Patel et al., 2016; Pereira et al., 2009; Vapnik, 1995). The linear SVM has only one parameter C, which determines the trade-off between the minimization of training error and misclassification penalty, was set to the default value (C = 1) for all cases. Finally, due to our limited number of samples, leave-one-out cross-validation (LOOCV) was used to quantify the classification performance (Wee et al., 2011).

The accuracy was defined as the proportion of samples correctly classified. The performance of the classifier was estimated with receiver operating characteristic (ROC) curves by calculating the area under the curve (AUC). The AUC of the ROC curve is thus often considered the most useful global marker of the diagnostic accuracy, with a larger AUC indicating a better classification power. we used the permutation test to cross-validate the AUC results because of the limited sample size in the present study. This entire process is repeated 5000 times, thereby yielding an unbiased estimation of the classification error rate. Unless specifically mentioned, the threshold for statistical significance was defined as P < 0.05.

**References**

Burges, C., 1998. A tutorial on support vector machines for pattern recognition. Data Min Knowl Disc, 121-167.

Chang, C., Lin, C., 2011. LIBSVM: a library for support vector machines. ACM Trans Intell Syst Technol (TIST), 2-27

Patel, M.J., Khalaf, A., Aizenstein, H.J., 2016. Studying depression using imaging and machine learning methods. Neuroimage Clin 10, 115-123.

Pereira, F., Mitchell, T., Botvinick, M., 2009. Machine learning classifiers and fMRI: a tutorial overview. Neuroimage 45, S199-209.

Vapnik, V., 1995. The nature of statistical learning theory. Springer, New York Inc;.

Wee, C.Y., Yap, P.T., Li, W., Denny, K., Browndyke, J.N., Potter, G.G., Welsh-Bohmer, K.A., Wang, L., Shen, D., 2011. Enriched white matter connectivity networks for accurate identification of MCI patients. Neuroimage 54, 1812-1822.

**Table S1. Brain regions of the main and interactive effects of APOE genotype and disease status on the normalized FOCA across all participants**

| Brain region | BA | Cluster size (mm3) | peak MNI coordinate (mm) | |  | Peak F score |
| --- | --- | --- | --- | --- | --- | --- |
|  |  |  | X | Y | Z |  |
| (1) Main effects of disease | |  |  |  |  |  |
| MFG.L/IFG.L | 46 | 1134 | -36 | 36 | 12 | 9.15 |
| STG.R/PCG.R | 41 | 2322 | 42 | -33 | 18 | 8.97 |
| MCG.L | 32 | 1458 | -15 | 18 | 21 | 7.80 |
| (2) Main effects of gene | |  |  |  |  |  |
| MFG.L | 10 | 810 | -30 | 42 | 9 | 16.35 |
| STG.R/MTG.R | 20 | 1539 | 54 | -18 | -3 | 14.97 |
| MOG.R | 18 | 837 | 27 | -102 | -3 | 18.21 |
| (3) Gene×disease interaction | |  |  |  |  |  |
| IFG.R | 46 | 2160 | 48 | 36 | 0 | 8.96 |
| FG.L | 10 | 3672 | -42 | 42 | 6 | 8.65 |
| PCUN.B | 7 | 1593 | -3 | -72 | 45 | 10.56 |
| IPL.L | 40 | 1593 | -30 | -54 | 54 | 8.93 |

Abbreviation: APOE, apolipoprotein E; LIns, left insula; LING.R, right lingual gyrus; MOG.R, right middle occipital gyrus; pMTG.L, left posterior middle temporal gyrus; SFG.L, left superior frontal gyrus; SFGmed.B, bilateral medial superior frontal gyrus; MCG.L, middle cingulate gyrus; MFG.R, middle frontal gyrus; THA.B, bilateral thalamus; SFGmed.L, left medial superior frontal gyrus.

**Table S2. Brain regions of the main and interactive effects of APOE genotype and disease status on the RIns network across all participants**

| Brain region | BA | Cluster size (mm3) | peak MNI coordinate (mm) | |  | Peak Z score |
| --- | --- | --- | --- | --- | --- | --- |
|  |  |  | X | Y | Z |  |
| (1) Main effects of gene |  |  |  |  |  |  |
| FFA.R | 37 | 1620 | 24 | -45 | -18 | 13.26 |
| MOG.R | 19 | 1323 | 36 | -90 | 12 | 11.83 |
| (2) Main effects of disease |  |  |  |  |  |  |
| SFGmed.R | 10 | 4428 | 15 | 48 | 6 | 7.28 |
| LING.L | 18 | 837 | -12 | -90 | -18 | 5.08 |
| (3) Gene×disease interaction |  |  |  |  |  |  |
| aMPFC.R | 11 | 3780 | 21 | 60 | -3 | 6.02 |

Abbreviation: APOE, apolipoprotein E; FFA.R, right fusiform areas; MOG.R, right middle occipital gyrus; SFGmed.R, right medial superior frontal gyrus; LING.L, left lingual gyrus; aMPFC.R, right anterior medial prefrontal cortex.


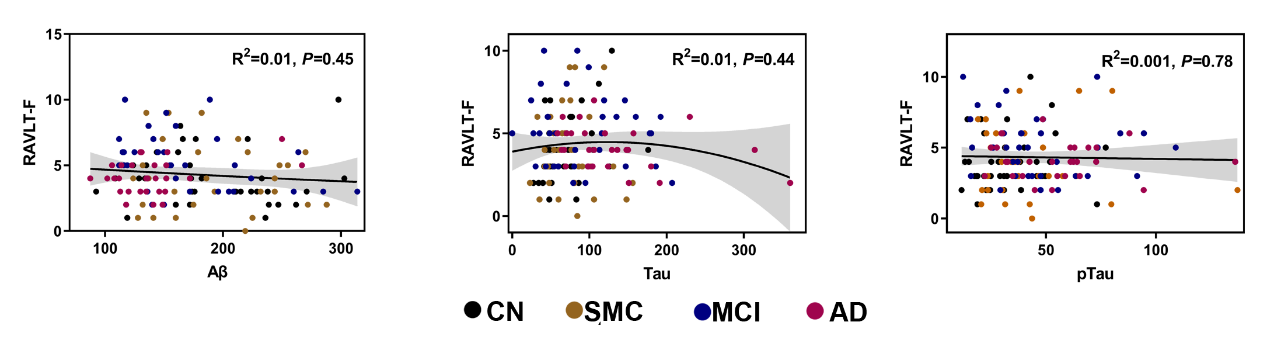


**Figure S1.** **Non-significant correlations were found between** **Aβ, pTau, Tau and RAVLT-F** **across the AD spectrum.** Abbreviation: Aβ, amyloid-β 1-42; Tau, total tau; pTau, phosphorylated tau; RAVLT-F, Rey auditory verbal learning test-forgetting; CN, cognitive normal; SMC, subjective memory complains; MCI, mild cognitive impairment; AD, alzheimer’s disease.

**
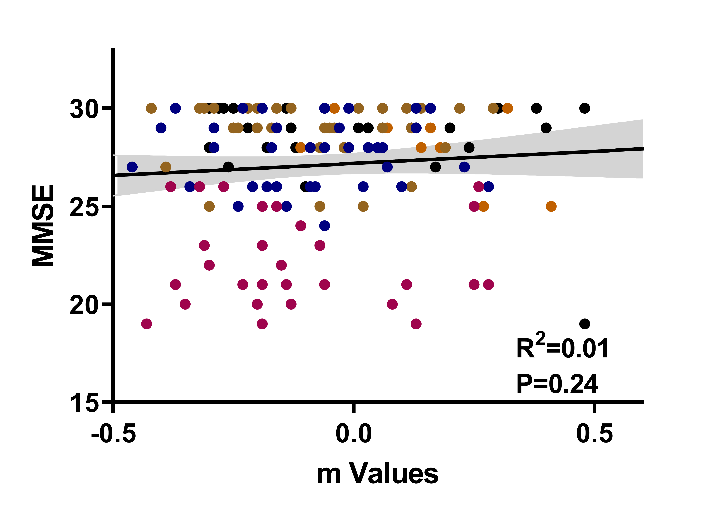
**


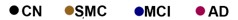


**Figure S2. Non-significant correlation was found between LIns-LING.R FC and MMSE across the AD spectrum**. Abbreviation: LIns, left insula; LING.R, right lingual gyrus; FC, functional connectivity; MMSE, Mini-Mental State Examination; CN, cognitive normal; SMC, subjective memory complains, MCI, mild cognitive impairment; AD, alzheimer’s disease.


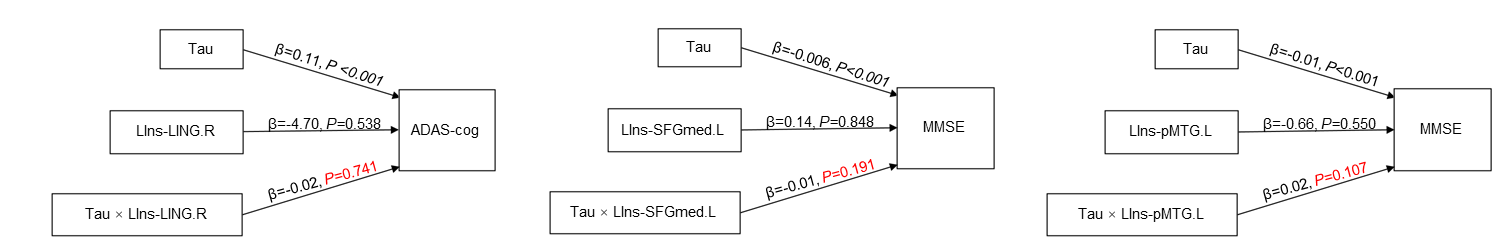


**Figure S3. Moderation analysis identified that the LIns-LING.R FC was not able to modulate the association between Tau and ADAS-cog, LIns-SFGmed.L and LIns-pMTG.L FCs could not regulate the association between Tau and MMSE across the AD spectrum (all P>0.05).** Abbreviation: Tau, total tau; LIns, left insula lobe; LING.R, right lingual gyrus; SFGmed.L, left medial superior frontal gyrus; pMTG.L, left posterior middle temporal gyrus; FC, functional connectivity; ADAS-cog, 13-item Alzheimer’s Disease Assessment Scale-Cognitive subscale; MMSE, Mini-Mental State Examination.


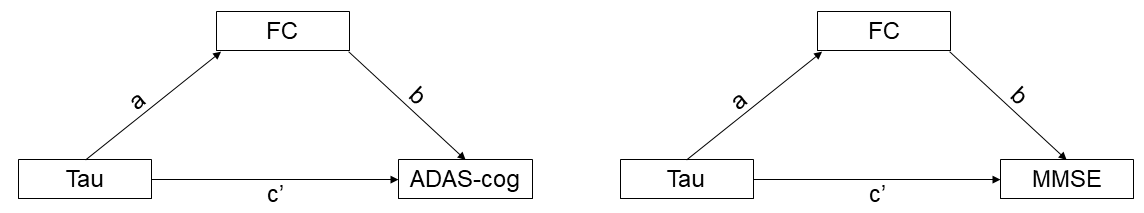


| IFC | 95% CI of a*b |  | IFC | 95% CI of a*b |
| --- | --- | --- | --- | --- |
| LIns-LING.R | [-0.001, 0.012] |  | LIns-LING.R | [-0.001, 0.003] |
| LIns-SFGmed.L | [-0.011, 0.004] |  | LIns-SFGmed.L | [-0.011, 0.004] |
| LIns-pMTG.L | [-0.001, 0.011] |  | LIns-pMTG.L | [-0.004, 0.001] |

**Figure S4. Mediation analysis** **identified that the IFCs were not able to modulate the association** **between Tau and ADAS-cog (or MMSE) across the AD spectrum.** The 95% CI for the path a*b crossed zero, the indirect effect of Tau on cognitive performance through the IFC was considered insignificant. Abbreviation: Tau, total tau; LIns, left insula lobe; LING.R, right lingual gyrus; SFGmed.L, left medial superior frontal gyrus; pMTG.L, left posterior middle temporal gyrus; IFC, insula functional connectivity; ADAS-cog, 13-item Alzheimer’s Disease Assessment Scale-Cognitive subscale; MMSE, Mini-Mental State Examination.

**
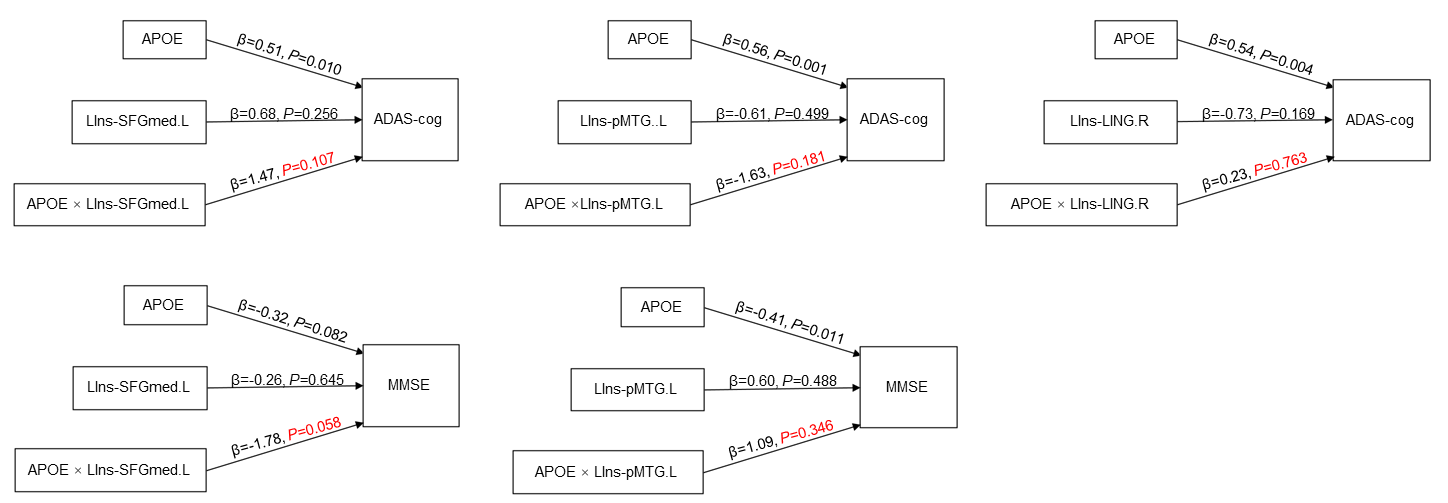
**

**Figure S5. Moderation analysis showed that the IFCs were not able to modulate the association between APOE genotype and cognitive performance across the AD spectrum (all P>0.05).** Abbreviation: APOE, apolipoprotein E; LIns, left insula lobe; SFGmed.L, left medial superior frontal gyrus; pMTG.L, left posterior middle temporal gyrus; LING.R, right lingual gyrus; IFC, insula functional connectivity; ADAS-cog, 13-item Alzheimer’s Disease Assessment Scale-Cognitive subscale; MMSE, Mini-Mental State Examination.
